# Autopsy detection of Pneumatosis Cystoides Intestinalis in a patient with end-stage renal failure from autosomal dominant polycystic kidney disease undergoing dialysis: a case report

**DOI:** 10.1093/omcr/omaf095

**Published:** 2025-11-26

**Authors:** Rei Sekiguchi, Kotaro Sugimoto, Akihiko Ozaki, Tomoro Kojima, Keigo Yoshida, Hiroaki Kawakami, Ashita Ono, Tomohiro Kurokawa, Kenji Gonda, Hiroaki Shimmura, Toyoaki Sawano

**Affiliations:** Clinical Training Center, Jyoban Hospital of Tokiwa Foundation, 57 Kaminodai, Jyobankamiyunagayamachi, Iwaki, Fukushima 972-8322, Japan; Department of Basic Pathology, Fukushima Medical University School of Medicine, 1 Hikarigaoka, Fukushima, Fukushima, 960-1295, Japan; Clinical Training Center, Jyoban Hospital of Tokiwa Foundation, 57 Kaminodai, Jyobankamiyunagayamachi, Iwaki, Fukushima 972-8322, Japan; Breast and Thyroid Cancer, Jyoban Hospital of Tokiwa Foundation, 57 Kaminodai, Jyobankamiyunagayamachi, Iwaki, Fukushima 972-8322, Japan; Clinical Training Center, Jyoban Hospital of Tokiwa Foundation, 57 Kaminodai, Jyobankamiyunagayamachi, Iwaki, Fukushima 972-8322, Japan; Clinical Training Center, Jyoban Hospital of Tokiwa Foundation, 57 Kaminodai, Jyobankamiyunagayamachi, Iwaki, Fukushima 972-8322, Japan; Department of Clinical Examination, Jyoban Hospital of Tokiwa Foundation, 57 Kaminodai, Jyobankamiyunagayamachi, Iwaki, Fukushima 972-8322, Japan; Department of Urology, Jyoban Hospital of Tokiwa Foundation, 57 Kaminodai, Jyobankamiyunagayamachi, Iwaki, Fukushima 972-8322, Japan; Department of Surgery, 57 Kaminodai, Jyobankamiyunagayamachi, Jyoban Hospital of Tokiwa Foundation, Iwaki City, Fukushima 972-8322, Japan; Breast and Thyroid Cancer, Jyoban Hospital of Tokiwa Foundation, 57 Kaminodai, Jyobankamiyunagayamachi, Iwaki, Fukushima 972-8322, Japan; Department of Urology, Jyoban Hospital of Tokiwa Foundation, 57 Kaminodai, Jyobankamiyunagayamachi, Iwaki, Fukushima 972-8322, Japan; Department of Surgery, 57 Kaminodai, Jyobankamiyunagayamachi, Jyoban Hospital of Tokiwa Foundation, Iwaki City, Fukushima 972-8322, Japan

**Keywords:** polycystic kidney, autosomal dominant, pneumatosis cystoides intestinalis, renal dialysis, autopsy, intestinal diseases

## Abstract

This report describes a rare case of pneumatosis cystoides intestinalis in a dialysis patient with autosomal dominant polycystic kidney disease, detected at autopsy. Pathological findings suggest a potential link between intestinal gas cyst formation and the structural fragility associated with polycystic kidney disease.

Autosomal Dominant Polycystic Kidney Disease (ADPKD) is a hereditary disorder characterized by cyst formation in kidney epithelial cells and other organs, including the liver and pancreas [1]. Although colonic diverticulosis is a recognized complication [2], Pneumatosis Cystoides Intestinalis (PCI)—a rare condition characterized by gas-filled cysts within the gastrointestinal wall, with an estimated incidence of 0.03%, and associated with factors such as bowel ischemia, chronic inflammation, and mechanical obstruction, presenting clinically from asymptomatic cases to severe complications such as bowel perforation [3]—has not previously been associated with ADPKD. This report highlights a unique case of PCI in a patient with ADPKD and end-stage renal failure on dialysis, underscoring a potential association between PCI and ADPKD.

The patient was a 60-year-old male diagnosed with end-stage renal failure due to ADPKD in 2008, who started dialysis thereafter. He also had a history of colonic diverticulitis in 2019 and succumbed to pulmonary thromboembolism in 2023. The patient experienced vomiting one day before death; however, bowel movements remained generally well-controlled with the prescribed medications.

Pathological autopsy revealed multiple diverticula in the small intestine, while the large intestine displayed severe PCI that spanned the entire length of the colon from the submucosa to the muscularis propria (Fig. 1A-C). The walls of the cyst are lined with macrophages, which is a typical histological finding of PCI (Fig. 1D).

**Figure 1:**
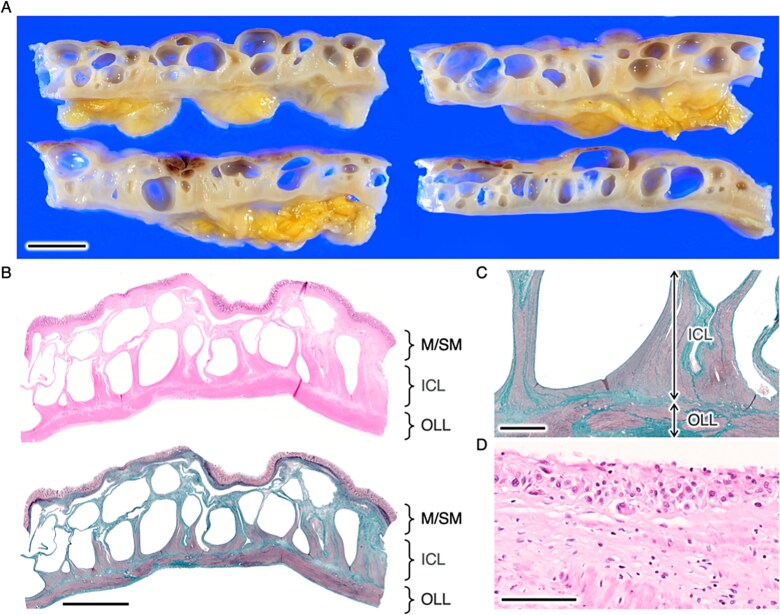
Gross (A) and microscopic (B-D) images of the colon. B (upper) and D are images of Hematoxylin and eosin staining, while B (bottom) and C are Masson trichrome. M, mucosa; SM, submucosa; ICL, inner circular layer; OLL, outer longitudinal layer. Bars, (A) 1 cm, (B) 5 mm, (C) 1 mm, and (D) 100 μm.

This case exhibited a multi-layered PCI with extensive emphysema reaching down to the internal circular muscle of the intrinsic muscular layer. Although no previous reports have linked ADPKD with PCI, it is hypothesized that PCI may have developed as a consequence of several ADPKD-related mechanisms. Potential mechanisms include systemic cyst formation, chronic inflammation, altered vascular supply, and connective tissue fragility associated with ADPKD [1], which may collectively weaken intestinal wall integrity and predispose it to gas infiltration and cystic changes. The patient’s history of colonic diverticulitis indicates underlying fragility of the bowel wall integrity [4].

Additionally, factors such as intestinal ischemia, chronic inflammation, and colonic diverticula should be considered in the development of PCI in this case [3]. Intestinal ischemia and chronic inflammation may weaken the intestinal wall, facilitating gas infiltration [3], while colonic diverticula can exacerbate wall compromise through localized inflammation and mechanical stress [4], though the patient’s diverticula may have developed in the context of ADPKD [1]. Combined with ADPKD-induced systemic changes, such as cyst formation and connective tissue fragility [1], these factors provide a plausible explanation for the extensive PCI observed. Further research is needed to clarify these relationships and explore the potential shared mechanisms between ADPKD and colonic diverticula.
